# AcFT promotes kiwifruit in vitro flowering when overexpressed and Arabidopsis flowering when expressed in the vasculature under its own promoter

**DOI:** 10.1002/pld3.68

**Published:** 2018-07-10

**Authors:** Sarah M. A. Moss, Tianchi Wang, Charlotte Voogd, Lara A. Brian, Rongmei Wu, Roger P. Hellens, Andrew C. Allan, Joanna Putterill, Erika Varkonyi‐Gasic

**Affiliations:** ^1^ The New Zealand Institute for Plant & Food Research Limited (Plant & Food Research) Mt Albert Auckland Mail Centre Auckland New Zealand; ^2^ School of Biological Sciences University of Auckland Auckland New Zealand; ^3^ Present address: The New Zealand Institute for Plant & Food Research Limited (Plant & Food Research) Palmerston North Palmerston North New Zealand; ^4^ Present address: Centre for Tropical Crops and Biocommodities Queensland University of Technology Brisbane Queensland Australia

**Keywords:** *Actinidia chinensis*, Florigen, *FLOWERING LOCUS T*, *FT*, Kiwifruit, promoter

## Abstract

Kiwifruit (*Actinidia chinensis*) has three *FLOWERING LOCUS T* (*FT*) genes, *AcFT*,* AcFT1,* and *AcFT2*, with differential expression and potentially divergent roles. *AcFT* was previously shown to be expressed in source leaves and induced in dormant buds by winter chilling. Here, we show that *AcFT* promotes flowering in *A. chinensis*, despite a short sequence insertion not present in other *FT*‐like genes. A 3.5‐kb *AcFT* promoter region contained all the regulatory elements required to mediate vascular expression in transgenic *Arabidopsis thaliana* (Arabidopsis). The promoter activation was initially confined to the veins in the distal end of the leaf, before extending to the veins in the base of the leaf, and was detected in inductive and noninductive photoperiods. The 3‐kb and 2.7‐kb promoter regions of *AcFT1* and *AcFT2*, respectively, demonstrated different activation patterns in Arabidopsis, corresponding to differential expression in kiwifruit. Expression of *AcFT* cDNA from the *AcFT* promoter was capable to induce early flowering in transgenic Arabidopsis in noninductive photoperiods. Further, expression of *AcFT* cDNA fused to the green fluorescent protein was detected in the vasculature and was also capable to advance flowering in noninductive photoperiods. Taken together, these studies implicate *AcFT* in regulation of kiwifruit flowering time and as a candidate for kiwifruit florigen.

## 1 INTRODUCTION

The transition to flowering is a highly regulated process in which developmental and environmental signals are integrated to ensure reproduction. *FLOWERING LOCUS T* (*FT*) (Kardailsky et al., 1999; Kobayashi, Kaya, Goto, Iwabuchi, & Araki, 1999) is the key floral integrator gene in *Arabidopsis thaliana* (Arabidopsis), encoding a major mobile flowering hormone ‘florigen’ (Chailakhyan, 1968; Zeevaart, 2008). *FT* is transcribed in the leaf vasculature, and the FT protein is subsequently transported in the phloem to the shoot apex, where it unloads and moves cell to cell to interact with the bZIP transcription factor FD and initiate flowering (reviewed in Abe et al., 2005; Andres & Coupland, 2012). The conservation of FT‐like protein role as florigen has been well established, but it has become clear that many FT‐like proteins perform other roles, including regulation of vegetative growth, storage organ differentiation, and fruit set (reviewed in Pin & Nilsson, 2012; Wickland & Hanzawa, 2015).

Previous studies have demonstrated that transcriptional regulation of *FT* was essential to ensure flowering time control in response to environmental conditions (Adrian et al., 2010; Takada & Goto, 2003) and more recent studies have begun to unravel the detailed mechanisms controlling FT movement and action (reviewed in Putterill & Varkonyi‐Gasic, 2016). In Arabidopsis and rice, the CONSTANS (CO)‐FT pathway is critical for photoperiodic regulation of *FT* transcription (Hayama, Yokoi, Tamaki, Yano, & Shimamoto, 2003; Samach et al., 2000; Suarez‐Lopez, 2001), but this pathway is not conserved in legumes (Putterill et al., 2013; Weller & Ortega, 2015) and little is known about activators of *FT* transcription in other species. Repressors such as Arabidopsis MADS‐box proteins FLOWERING LOCUS C (FLC) and SHORT VEGETATIVE PHASE (SVP) bind to the CArG box motifs in the first intron and the promoter region of *FT* (Lee et al., 2007; Li et al., 2008; Searle et al., 2006) to repress flowering, but this role may not be universal. Further layers of complexity in regulation of FT include chromatin‐mediated changes (Adrian et al., 2010; He, 2015; Kotake, Takada, Nakahigashi, Ohto, & Goto, 2003) and posttranslational regulation (Kim et al., 2016). It is unclear whether these mechanisms are conserved across species, but it has been established that FT‐like proteins interact with conserved protein partners including FD homologs (Abe et al., 2005; Pnueli et al., 2001; Wigge et al., 2005) and other transcription factors (Ho & Weigel, 2014; Mimida et al., 2011; Niwa et al., 2013).

The long life span, large size, coexistence of vegetative, and floral fate on the same plant, combined with the lack of natural mutants and lengthy transformation, have resulted in a much slower progress in studies of *FT* homologs in woody perennial plants, where many questions about gene diversification and florigen function remain unresolved (Putterill & Varkonyi‐Gasic, 2016). However, the importance of FT in regulation of flowering time was demonstrated by expression of endogenous or heterologous *FT* genes, which dramatically reduced the juvenile period and provided a means to accelerate breeding in trees (Böhlenius et al., 2006; Endo et al., 2005; Hsu, Liu, Luthe, & Yuceer, 2006; Klocko et al., 2016; Kotoda et al., 2010; Song, Walworth, Zhao, Jiang, & Hancock, 2013; Srinivasan, Dardick, Callahan, & Scorza, 2012; Wenzel, Flachowsky, & Hanke, 2013), but these approaches have often proven to be unreliable (Zhang et al., 2010). Most of the studies utilized strong overexpression, which generally induced very early flowering and failed to distinguish between *FT* paralogs. However, in some cases overexpression of endogenous *FT* genes did not result in precocity, despite their ability to promote flowering in model annuals. Instead, they affected dormancy and leaf senescence in apple (Freiman et al., 2015) or shoot vigor in kiwifruit (Varkonyi‐Gasic et al., 2013), suggesting roles besides flowering time control. Moderate overexpression comparable to normal peak expression revealed functional divergence among *FT* paralogs to determine reproductive onset and vegetative growth in poplar (Hsu et al., 2011). However, for a large number of *FT*‐like genes from woody perennial species, the function, regulation, and modes of action remain largely unknown.

In this study, we are focusing on *FT*‐mediated regulation of growth and flowering in kiwifruit. Kiwifruit are deciduous woody perennial vine species of the genus *Actinidia*, recently domesticated as a horticultural crop, with increasing importance but very short history of breeding and limited genetic resources (Datson & Ferguson, 2011). *Actinidia* are characterized by a juvenile unproductive period and a temperate flowering phenology. The growth cycle is spread between two seasons, interrupted by winter dormancy. In the first season, meristems with a potential to differentiate flowers are initiated in the axils of leaf primordia, within the lateral buds of developing shoots (Varkonyi‐Gasic et al., 2011; Walton, Fowke, Weis, & Mcleay, 1997; Walton, Podivinsky, & Wu, 2001). In natural conditions, autumn short days and cooler temperatures induce growth cessation and these lateral buds become dormant (Lionakis & Schwabe, 1984). Accumulation of chilling during the winter dormancy period is essential to resume growth and flowering in spring (Brundell, 1976). Inflorescences develop in the lower leaf axils of shoots emerging after the chilling period, from the preestablished meristems. Photoperiod has no clear role in regulation of flowering in spring (Snelgar, Clearwater, & Walton, 2007), but insufficient chilling results in sporadic budbreak, low flower numbers, and low fruit yield.

Recently, we identified and characterized three *FT* genes from a commercial kiwifruit species *Actinidia chinensis*, designated *AcFT*,* AcFT1,* and *AcFT2* (Varkonyi‐Gasic et al., 2013; Voogd, Brian, Wang, Allan, & Varkonyi‐Gasic, 2017). They all promoted flowering when expressed in Arabidopsis, but were differentially expressed in kiwifruit, suggesting that they evolved to perform distinct roles. *AcFT* expression domains included leaves and dormant buds, implicating it in regulation of budbreak and flowering. In contrast, *AcFT1* and *AcFT2* were fully excluded from the dormant bud. *AcFT1* was detected in the tip and small apical leaves of actively growing shoots, and *AcFT2* was detected in large basal leaves. Strong ectopic expression of both *AcFT1* and *AcFT2* promoted in vitro flowering in *A. chinensis*, indicating that AcFT1 and AcFT2 may function as flowering activators in kiwifruit, although their expression patterns could not fully explain this role. Conversely, ectopic overexpression of the third *Actinidia FT* gene, *AcFT*, did not cause another species of kiwifruit, *A. eriantha*, to flower more rapidly. Therefore, it remained unclear whether *AcFT* was a kiwifruit flowering gene.

Here, we ectopically overexpressed *AcFT* in *A. chinensis* to establish whether its strong ectopic expression promoted flowering. Next, we studied the expression, function, and localization of *AcFT* using reporter gene fusions and gene expression constructs driven by the *AcFT* promoter. These studies were performed in Arabidopsis to circumvent the inefficient transformation, large size and long juvenility of kiwifruit and to take advantage of absence of endogenous *FT* in noninductive photoperiods. Constructs driven by the *AcFT1* and *AcFT2* promoters were also included in the study and the *SUCROSE TRANSPORTER 2* (*SUC2*) promoter (Stadler, Lauterbach, & Sauer, 2005), which is specific to the phloem companion cells of the major veins and commonly used to determine whether genes encode florigen‐like activity, was included in control experiments.

## 2 MATERIALS AND METHODS

### 2.1 Amplification of genes and vector construction

The full‐length *AcFT* coding sequence was amplified from kiwifruit *A. chinensis* var. *chinensis* ‘Hort16A’ cDNA, cloned into pUC19 vector and verified by sequence analysis as previously described (Varkonyi‐Gasic et al., 2013). After addition of attB sites by PCR, it was recombined using Gateway into pDONR221, verified by sequence analysis and recombined into pHEX2 (Hellens et al., 2005), placing the cDNA between the CaMV *35S* promoter and the *ocs* 3′ transcriptional terminator. The AcFT* sequence with added attL sites was synthesized by GenScript (http://www.genscript.com) and recombined into pHEX2 as above. The resulting plant transformation vectors were transformed into *Agrobacterium tumefaciens* strain EHA105 by electroporation.

The 3.5‐kb sequence upstream of the *AcFT* translation start site (GenBank accession KJ439053) was obtained using inverse PCR (Ochman, Gerber, & Hartl, 1988) from kiwifruit ‘Hort16A’ genomic DNA digested with HindIII and circularized using T4 DNA ligase (Invitrogen, Life Science Technologies, Carlsbad, CA). For the cloning of *AcFT1* and *AcFT2* promoter sequences, multiple primers were designed based on the draft *Actinidia* genome (Huang et al., 2013) and used for amplification from the kiwifruit ‘Hort16A’ genomic DNA template. The 3.0‐ and 2.7‐kb sequence upstream of the *AcFT1* and *AcFT2* translation start sites, respectively (GenBank accession numbers KX611592 and KX611593) were chosen for subsequent study as the longest promoter fragments. All promoters included the 5′UTR sequences of unknown size. They were cloned into pGEM‐T (Promega, Madison, WI), verified by sequence analysis, reamplified to include appropriate restriction sites and subsequently cloned to generate fusion constructs.

The 1.8‐kb fragment of the Arabidopsis *FT* promoter was amplified from Col‐0 genomic DNA and the Arabidopsis *FT* coding region was amplified from Col‐0 seedling cDNA. The vascular‐specific *SUC2* promoter, *AcFT1* and *AcFT2* cDNA (Voogd et al., 2017) were previously described. The promoter‐less *GUS* and *pro35S:GUS* vector controls were previously constructed from pHEX2 (Hellens et al., 2005). pHEX2S:GFP derivative of pHEX2, designed previously to include the *35S* promoter‐driven sequence encoding GFP(S65T) (Heim, Cubitt, & Tsien, 1995), was used to generate GFP fusion constructs and pGreenII 0800‐LUC vector (Hellens et al., 2005) was used to generate LUC fusions. Oligonucleotide primer sequences used for amplification and as linkers are presented in Table S1 and generation of constructs used in this study in Table S2. All the resulting plant transformation vectors were transformed into *Agrobacterium tumefaciens* strain GV3101 by electroporation.

### 2.2 Plant material and transformation

*Agrobacterium*‐mediated transformation of *A. chinensis* ‘Hort16A’ was as previously described (Voogd, Wang, & Varkonyi‐Gasic, 2015; Wang, Atkinson, & Janssen, 2007), using *A. tumefaciens* strain EHA105. *Agrobacterium‐*mediated transformation of Arabidopsis Col‐0 (Clough & Bent, 1998; Martinez‐Trujillo, Limones‐Briones, Cabrera‐Ponce, & Herrera‐Estrella, 2004) was performed using *A. tumefaciens* strain GV3101.

### 2.3 Plant growth conditions and sampling

Seeds of transgenic Arabidopsis plants were selected on half‐strength Murashige and Skoog (½ MS) medium supplemented with kanamycin and placed in a growth room under a long‐day (LD, 21°C, 16/8‐hr light/dark) or short‐day (*SD*, 21°C, 8/16 hr light/dark) regime. Subsequently, plants were grown in soil using a standard potting mix in LD and SD regime. Because *proAcFT* is most highly active during early stages of development, expression analysis in kanamycin‐resistant lines was performed 7 days after germination (DAG) for transgenic Arabidopsis, using 10 T2 kanamycin‐resistant Arabidopsis seedlings of each line. All sampling was performed at the end of the light cycle to minimize variation.

### 2.4 RNA extraction and expression studies

Total RNA from Arabidopsis was isolated using the Trizol reagent (Invitrogen) and from kiwifruit using the Spectrum Plant Total RNA Kit (Sigma‐Aldrich, St. Louis, MO, USA). Reverse transcription (RT) was performed using a 1 μg aliquot of RNA treated with DNase I (Invitrogen), an oligo(dT) primer and the SuperScript III reverse transcriptase (Invitrogen). Quantifications using real‐time PCR were performed with the FastStart DNA Master SYBR Green I mix (Roche Diagnostics, Mannheim, Germany) using the LightCycler 1.5 instrument and the LightCycler Software version 4 (Roche Diagnostics). Amplification was carried out using a 10^−3^ dilution of the cDNA template, with an initial denaturing step at 95°C for 5 min, then 40–50 cycles of 95°C for 5 s, 60°C for 5 s, and 72°C for 10 s. A nontemplate control was included in each run. Oligonucleotide primers (Table S1) were designed to produce amplification products of 100–150 nucleotides. The specificity of primer pairs was confirmed by melting curve analysis of PCR products and agarose gel electrophoresis followed by sequence analysis. The expression was normalized to Arabidopsis *ACT2* (At3 g18780) and kiwifruit *A. chinensis* var. *deliciosa ACTIN* (GenBank accession FG403300).

### 2.5 Histochemical localization of GUS activity

Detection of GUS activity was performed according to Jefferson (1987) with some modifications. Briefly, whole seedlings or excised tissue were soaked in the staining solution (50 mM NaPO_4_ pH7.0, 0.05% X‐Gluc (Gold Biotechnology, St. Louis, MO), 0.2% dimethylformamide, 10% methanol and 0.1% Tween 20) for 24 hr at 37°C, rinsed twice with water followed by 50% ethanol and soaked in ethanol to remove chlorophyll, stored in fresh ethanol and photographed. Whole seedlings and plant organs or hand sections of tissue were used for photographing.

To analyze transgenic Arabidopsis plants carrying the GUS fusion constructs, a minimum of 10 independent transgenic lines for each GUS fusion construct were screened to determine if GUS activity could be detected, but allowing plants to grow and produce seed. For the detailed screen, progeny of at least three independent lines were used for staining at six time points during development in LD conditions, to represent the cotyledon stage (7 DAG), rosette leaf development (4‐5 rosette leaves, ~12 DAG), floral transition (9‐11 rosette leaves, ~26 DAG), early and late flower development (inflorescence stem 10–20 cm long), and late maturity (multiple inflorescences, silique development). For the first four time points, whole seedlings on ½ MS medium were used. For the last two time points, staining was performed using aerial parts of plants grown in soil. Progeny of the same lines were also grown in SD regime and stained at corresponding time points.

### 2.6 Promoter transactivation assays

*Nicotiana benthamiana* plants were grown in LD conditions, and transient leaf assays carried out as described in Hellens et al. (2005). Assays were performed using three biological replicates and four technical replicates. Sampling of infiltrated leaves was performed 3 days post infiltration. The quantification was performed using the Dual‐Glo Luciferase Assay System (Promega) and the firefly luciferase (LUC) and *Renilla* luciferase (REN) luminescence were measured using the Orion Microplate Luminometer and Simplicity software (Berthold Detection Systems). The promoter activity was quantified as the LUC/REN chemiluminescence ratio.

### 2.7 Imaging

Photography of whole seedlings or plant organs was performed using a Nikon D80 DSLR with 60 mm Micro‐Nikkor lens. GFP activity was examined using the Leica MZ FLIII stereo fluorescence microscope with a GFP2 and GFP3 filter (Leica Microsystems, Wetzlar, Germany) equipped with the Infinity2 camera (Lumenera, Ottawa, ON, Canada).

## 3 RESULTS

### 3.1 Ectopic expression of *AcFT* in *A. chinensis* promotes in vitro flowering

**Figure 1:**
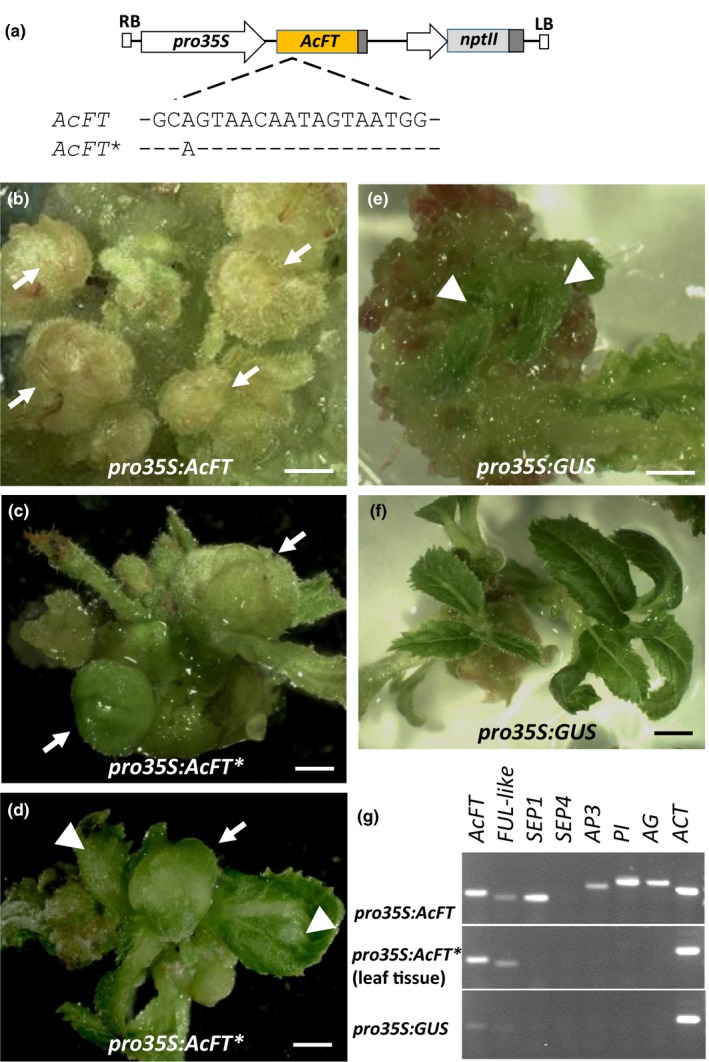
Transgenic kiwifruit flowering in vitro. (a) Schematic diagram of the constructs of the full‐length *AcFT* coding sequence (*pro35S:AcFT*) and a modified *AcFT* coding sequence designed to exclude the unusual region (pro35S:AcFT*). (b, c) Proliferation of in vitro flowers within 2 months from initiation of transformation with both constructs. (d) Flower and leaf development in pro35S:AcFT* plantlets. (e) Leaf initials in the control transformation. (f) Vegetative control plantlets. (g) RT–PCR of *AcFT* and kiwifruit *FRUITFULL‐like* (*FUL‐like*), *SEPALLATA1* (*SEP1*), *SEPALLATA4* (*SEP4*), *APETALA3* (*AP3*), *PISTILLATA* (*PI*), *AGAMOUS* (*AG*), and *ACTIN* (*ACT*). Flower and leaf tissue are indicated by arrows and arrowheads, respectively. Bars, 1 mm (b, e), 2 mm (c, d, f)

The *AcFT* coding sequence contains an unusual region, not identified in any other *FT* gene, giving rise to an insertion of 6 amino acids close to the N‐terminus (Figure S1). Transformation of *A. chinensis* was therefore initiated using two constructs, the full‐length *AcFT* coding sequence and a modified *AcFT* coding sequence designed to exclude the unusual region, both driven by the cauliflower mosaic virus (CAMV) *35S* promoter (Figure 1a). Proliferation of flower‐like initials which never differentiated further and died within 2 months of the transformation experiment was observed with the *AcFT* construct (*pro35S*:*AcFT*; Figure 1b). The modified *AcFT* construct (*pro35S*:AcFT*) gave rise to in vitro flowers (Figure 1c) and very early flowering plantlets (Figure 1d). In contrast, development of leaf‐like structures (Figure 1e) followed by normal vegetative growth (Figure 1f) were seen in control (*pro35S*:*GUS*) experiments. To confirm floral identity of *pro35S:AcFT* flower‐like initials, amplification of flower‐specific MADS‐box transcripts (Varkonyi‐Gasic et al., 2011) and *SEPALLATA4* (*SEP4*) abundant during fruit development (Richardson et al., 2011) was performed. The presence or absence of kiwifruit *SEP1*,* APETALA3* (*AP3*), *PISTILLATA* (*PI*) and *AGAMOUS* (*AG*) transcripts revealed floral and vegetative fates in appropriate tissues (Figure 1g). Therefore, both constructs strongly promoted flowering in vitro and the slightly reduced activity after removal of the unusual small insertion in AcFT* resulted in distinguishable flower organ development, but neither construct gave rise to viable plants.

### 3.2 The 3.5‐kb sequence upstream of the *AcFT* translation start site was sufficient for specific vascular expression in Arabidopsis

**Figure 2:**
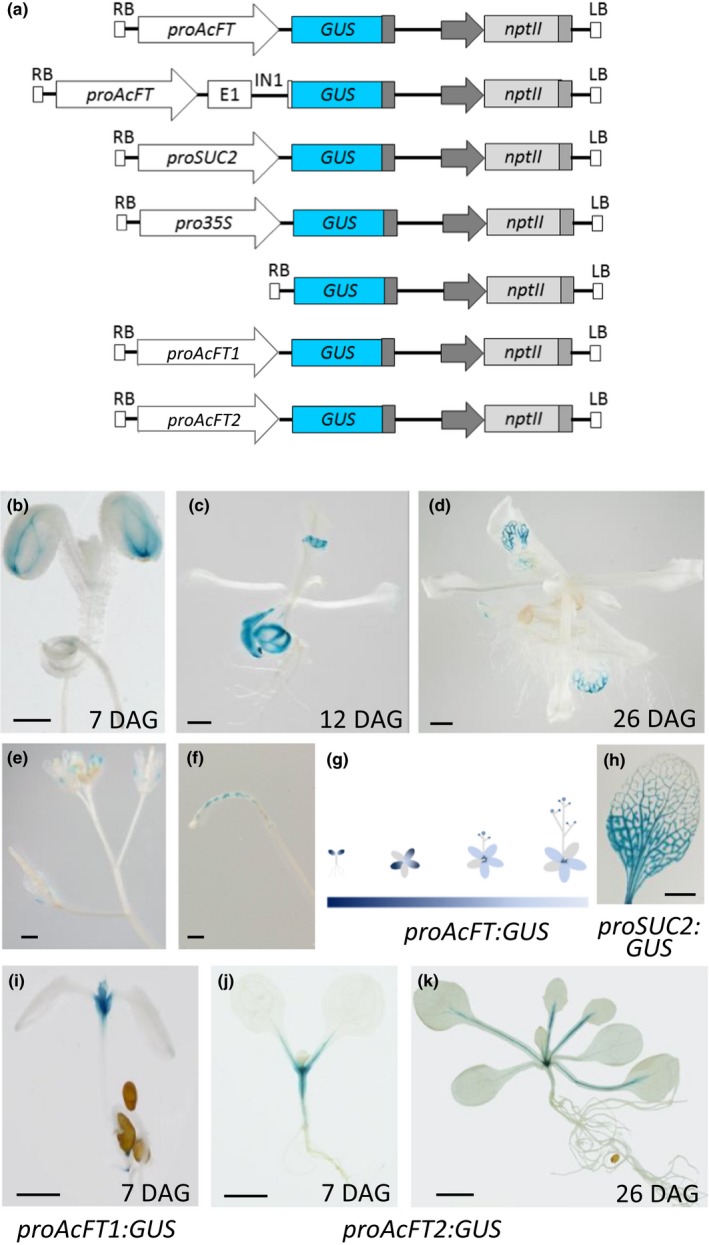
Temporal and spatial regulation of the *AcFT* promoter (*proAcFT*) in transgenic Arabidopsis in long‐day conditions (LD). (a) Promoter fusion constructs in the binary vector: 3.5‐kb sequence upstream of the *AcFT* translation start sites (*proAcFT*), a fragment containing *proAcFT*,* AcFT* first exon (E1), first intron (IN1) and the first eight codons of the second exon, *SUCROSE TRANSPORTER 2* (*SUC2*) promoter (*proSUC2*), CaMV *35S* promoter (*pro35S*), 3‐kb sequence upstream of the *AcFT1* translation start sites (*proAcFT1*), and 2.7‐kb sequence upstream of the *AcFT2* translation start sites (*proAcFT2*) fused with *uidA* (*GUS*) reporter gene. (b–f) Histochemical localization of GUS activity in transgenic *proAcFT*:*GUS* Arabidopsis grown in long‐day conditions (LD) during the cotyledon stage (b), rosette leaf development (c), and floral transition (d), in flowers (e), and in siliques (f). (g) Schematic summary of the position and intensity of GUS staining. (h) Histochemical localization of GUS activity in transgenic *proSUC2*:*GUS* Arabidopsis leaf. (i–k) Histochemical localization of GUS activity in transgenic *proAcFT2*:*GUS* Arabidopsis during the cotyledon stage (i, j) and at floral transition (k). Bars, 1 mm; DAG, days after germination

To study the regulation of *AcFT* expression, a 3.5‐kb sequence upstream of the *AcFT* translation start site was cloned from *A. chinensis* genomic DNA and designated *proAcFT*. To evaluate the activity of this promoter fragment, a transcriptional fusion with the reporter gene *uidA* (*GUS*) was prepared. A translational fusion including the *AcFT* first exon, first intron and the first eight codons of the second exon was also evaluated to examine whether regulatory regions exist in the first intron. Constructs containing the constitutive CaMV *35S* and the vascular‐specific *SUC2* promoters fused to the *GUS* gene were used as positive controls and the promoter‐less *GUS* construct was used as a negative control. In addition, to compare the regulation of *AcFT* expression with the other two kiwifruit *FT* genes, a 3‐kb and 2.7‐kb sequence upstream of the *AcFT1* and *AcFT2* translation start sites, respectively were isolated from *A. chinensis* genomic DNA, designated *proAcFT1* and *proAcFT2*, and fused with the *GUS* gene (Figure 2a).

*GUS* expression driven by the *proAcFT* fragment (*proAcFT:GUS*) was developmentally regulated and restricted to the vascular tissue of transgenic Arabidopsis. Initially, strong expression was detected at the distal end of the cotyledons (Figure 2b), followed by expression throughout the vasculature of cotyledons and veins in the distal half of developing leaves (Figure 2c,d). At later stages of development, the signal was detected in the veins in the distal half of sepals (Figure 2e) and weak staining was observed in the phloem of the vasculature at the base of rosette leaves. In some lines, GUS staining was detected in siliques (Figure 2f). The promoter activity in the vasculature during transgenic Arabidopsis development is summarized in a schematic (Figure 2g). GUS signal was not detected in the shoot apical meristem, leaf primordia, hypocotyls or roots. A similar pattern was obtained with the construct that included the first intron (Figure S2). The control CaMV *35S* provided the expected constitutive activity and the *SUC2* promoter demonstrated strong vascular expression throughout development (Figures 2h, S2).

In contrast to *proAcFT*,* GUS* expression driven by the *proAcFT1* fragment was observed in the shoot tip of transgenic Arabidopsis plants (Figure 2i), while *GUS* expression driven by the *proAcFT2* fragment was detected in the vascular tissue of the cotyledon petiole and the hypocotyl (Figure 2j), followed by expression in the petioles and midribs at the basal end of the leaf (Figures 2k, S3). Therefore, different expression patterns were demonstrated for each kiwifruit *FT* gene, similar to their differential expression in kiwifruit (Varkonyi‐Gasic et al., 2013; Voogd et al., 2017), but only the *GUS* expression driven by the *proAcFT* fragment was following the basipetal leaf development pattern, closely resembling the pattern of the Arabidopsis *FT* promoter (Adrian et al., 2010).

### 3.3 The *AcFT* promoter is active under the noninductive photoperiod in transgenic Arabidopsis

**Figure 3:**
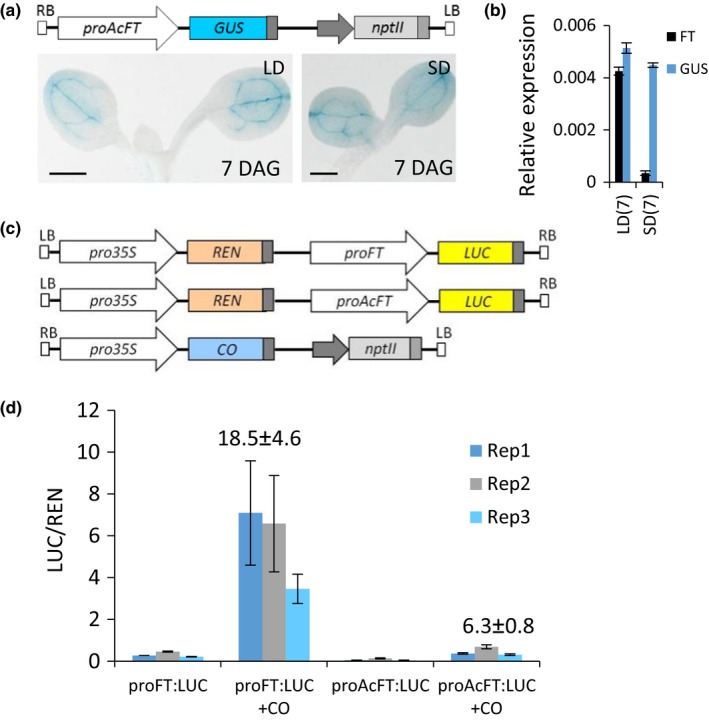
The *AcFT* promoter is not strongly regulated by photoperiod in transgenic Arabidopsis. (a) Histochemical localization of GUS activity in cotyledons of transgenic *proAcFT*:*GUS* Arabidopsis grown in inductive LD and noninductive SD. Bars, 1 mm. (b) Relative expression ± *SE* of Arabidopsis *FT* and *GUS* in transgenic *proAcFT*:*GUS* Arabidopsis grown in LD and SD for 7 days. The sampling was performed at the end of the light phase. (c) Schematic diagram of constructs used in *Agrobacterium*‐mediated transient promoter transactivation assays performed in *Nicotiana benthamiana*. Fusion constructs of promoters of Arabidopsis *FT* (*proFT*) and kiwifruit *AcFT* (*proAcFT*) with firefly luciferase (*LUC*) gene were coinfiltrated with Arabidopsis *CONSTANS* (*CO*) driven by the *35S* promoter. *Renilla* luciferase (*REN*) driven by *35S* promoter is used to normalize the assays. (d) *CO*‐mediated induction of Arabidopsis *FT* and *AcFT* promoters measured as LUC/REN ratio. The average values of fold change for all three biological replicates are indicated above. Error bars represent standard deviations for four technical replicates

In Arabidopsis, *FT* is expressed at a much higher level in long‐day (LD) than in short‐day (SD) conditions due to direct activation by CO (Samach et al., 2000). In contrast, the expression of *GUS* transcript and intensity of GUS staining of the *proAcFT:GUS* transgenic plants grown in different photoperiods were similar (Figure 3a,b), suggesting that it is not regulated by the Arabidopsis photoperiodic “switch,” CO. To further investigate, transactivation studies of a firefly luciferase (*LUC*) gene under the control of the Arabidopsis *FT* or *AcFT* promoters were performed in *Nicotiana benthamiana*. A 1.8‐kb fragment upstream of the Arabidopsis *FT* translation start site was isolated and a transcriptional fusion with *LUC* was generated. A transcriptional fusion of the 3.5‐kb *AcFT* promoter fragment with *LUC* was also prepared. Both constructs contained the *Renilla* luciferase gene (*REN*) driven by the *35S* promoter fragment to normalize the luminescence recording (Figure 3c). The *Agrobacterium* carrying appropriate promoter constructs was infiltrated into *N. benthamiana* leaves on plants growing in LD photoperiods, to determine basal luminescence and coinfiltrated with *Agrobacterium* carrying a construct of Arabidopsis *CO* cDNA under the control of the *35S* promoter (*pro35S*:*CO*; Figure 3c) to determine the effect of CO on *LUC* expression. Coinfiltration with *pro35S*:*CO* resulted in a significant increase in luminescence with the Arabidopsis *FT* promoter, but only a small increase with *proAcFT* (Figure 3d). We conclude that the *proAcFT* fragment is largely independent of photoperiodic regulation in transgenic Arabidopsis and is active in conditions when endogenous *FT* is not expressed.

### 3.4 *AcFT* can promote flowering in Arabidopsis when expressed under its own promoter

**Figure 4:**
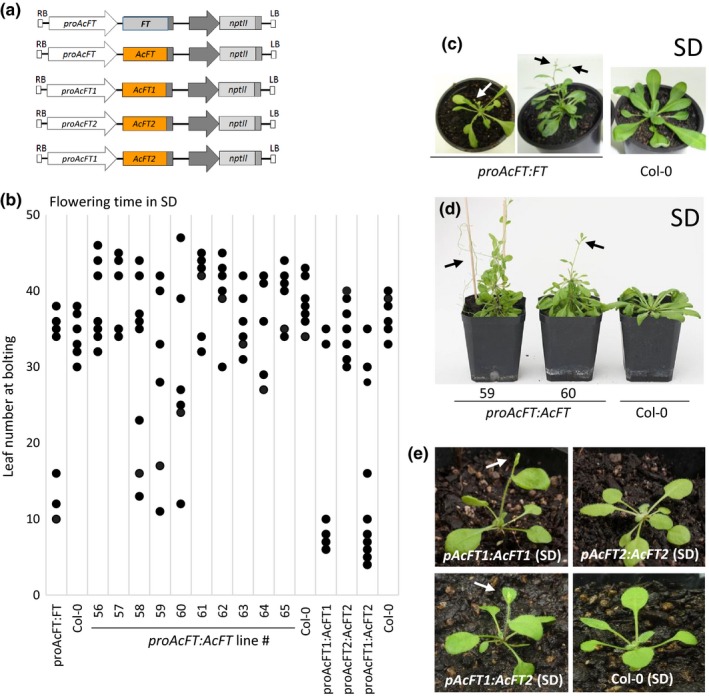
Expression of an *FT* gene from the *AcFT* and *AcFT1* promoters is sufficient for flowering in noninductive conditions in transgenic Arabidopsis. (a) Fusion constructs of kiwifruit (*Ac*) *FT* promoters with Arabidopsis *FT* and kiwifruit (*Ac*) *FT* cDNAs. (b) The range of flowering time in Arabidopsis grown in SD conditions. Flowering time was recorded as the total leaf number when the primary inflorescence stems were 0.2 cm long. Each dot represents the flowering time of at least one line; multiple lines with the same flowering time are presented as single dots. (c) Early bolting and growth of aerial rosettes in transgenic *proAcFT*:*FT* Arabidopsis in SD. (d) Early bolting and growth of aerial rosettes in transgenic *proAcFT*:*AcFT* Arabidopsis in SD. (e) Early flowering in *proAcFT1*:*AcFT1* and *proAcFT1*:*AcFT2* Arabidopsis in SD. Flowers and siliques are indicated by arrows

The activity in SD provided the opportunity to evaluate if *FT* expression driven by the *proAcFT* fragment was sufficient to supplement for the lack of endogenous *FT* in noninductive conditions. A transcriptional fusion with Arabidopsis *FT* cDNA under control of *proAcFT* (*proAcFT:FT*; Figure 4a) was introduced into Arabidopsis Col‐0. Three out of seven lines flowered early in SD conditions, after the plants produced between 10 and 16 leaves, in contrast to >30 in control lines (Figure 4b), demonstrating that expression of *FT* from *proAcFT* could induce flowering under noninductive conditions, although the flowers often aborted and some aerial rosette growth was observed (Figure 4c).

Next, to evaluate if the *AcFT* coding sequence under control of its own promoter (*proAcFT:AcFT*; Figure 4a) may also be sufficient to promote flowering, ten *proAcFT:AcFT* lines in Col‐0 were generated. To evaluate if they can promote flowering in absence of endogenous *FT* under noninductive conditions, kanamycin‐resistant progeny expressing *AcFT* (Figure S4) was monitored. Progeny of three lines showed early bolting in SD (Figure 4b), followed by growth of numerous aerial rosettes (Figure 4d). We conclude that *AcFT* under its own promoter can activate flowering in Arabidopsis, but may not be sufficient to maintain flowering in noninductive conditions.

We also evaluated if *AcFT1* and *AcFT2* under the control of their own promoters affect flowering, using *proAcFT1:AcFT1* and *proAcFT2:AcFT2* constructs transformed into Arabidopsis (Figure 4a, S4). Very early flowering in SD conditions was recorded for some *proAcFT1:AcFT1*, without reversion to vegetative growth, but not for *proAcFT2:AcFT2* plants (Figure 4b,e). Expression of *AcFT2* under control of *proAcFT1* (*proAcFT1:AcFT2*) was also sufficient for very early flowering in SD conditions (Figure 4b,e). Taken together, the data show that expression of an *FT* cDNA from *proAcFT* in leaf veins and *proAcFT1* in the shoot tip may be sufficient for promotion of flowering, and in case of *proAcFT1* maintenance of floral fate, while the *AcFT2* promoter fragment used in this study did not show such effect.

### 3.5 Vascular expression of *AcFT:GFP* promotes flowering in Arabidopsis

**Figure 5:**
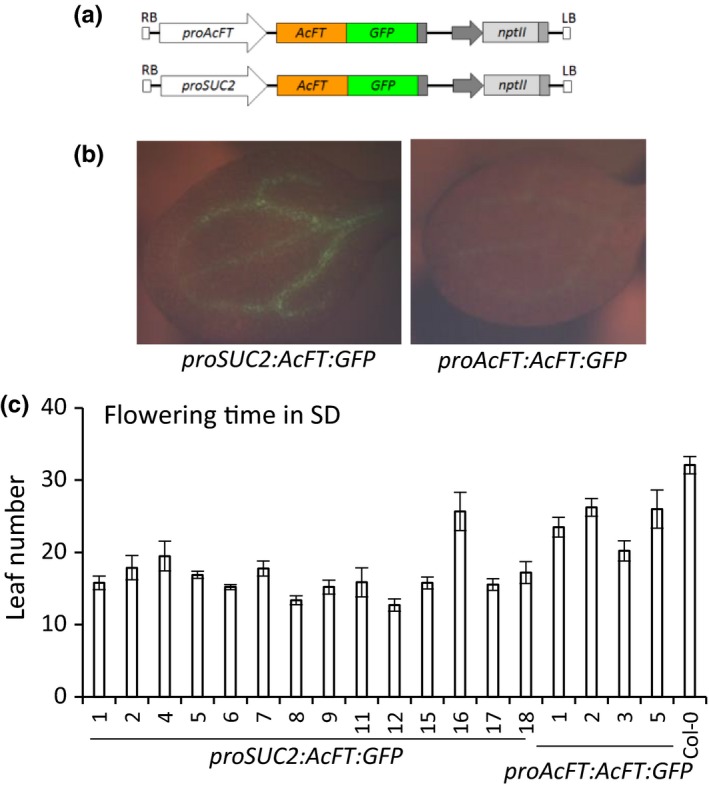
Expression of *AcFT:GFP* in the phloem promotes flowering in noninductive conditions in transgenic Arabidopsis. (a) Fusion constructs with *GFP* reporter gene used in this study. (b) GFP (green fluorescence) detected in cotyledons of transgenic Arabidopsis. (c) Early flowering in transgenic Arabidopsis. The minimum of eight T2 kanamycin‐resistant lines were monitored for flowering time, which was recorded as the total leaf number when the primary inflorescence stems were 0.2 cm long and presented as means ± *SE*. ANOVA *p*‐value of ≤ 0.01 was used to determine statistical significance for presented lines

To study the AcFT protein localization, *AcFT* cDNA fused to the green fluorescent protein (GFP) coding sequence (*AcFT:GFP*) was placed under control of *AcFT* and *SUC2* promoters (Figure 5a) and transformed into Arabidopsis. The expected vascular GFP activity was observed in cotyledons of *proSUC2:AcFT:GFP* transgenic lines and plants flowered significantly earlier in SD (Figure 5b,c), confirming functionality and potential mobility of the fusion AcFT:GFP protein in Arabidopsis. Much weaker green florescence was detected in the veins in cotyledons of *proAcFT:AcFT:GFP* transgenic lines (Figure 5b), but earlier flowering compared to controls was recorded (Figure 5c), confirming flower‐promoting activity of *AcFT:GFP* expressed from *proAcFT*.

## 4 DISCUSSION

### 4.1 *AcFT* promotes floral fate in kiwifruit *A. chinensis*

In this study, we investigated the ability of *AcFT* to promote flowering in kiwifruit and Arabidopsis and characterized its expression using fusion constructs in Arabidopsis. Previous studies implicated *AcFT* in regulation of floral transition, but this was not confirmed by our initial transgenic studies in kiwifruit *A. eriantha*. In addition, *AcFT* was also expressed in developing flowers and fruit, suggesting additional roles in regulation of floral fate and fruit development (Varkonyi‐Gasic et al., 2013).

Here we overexpressed *AcFT* in *A. chinensis* and obtained very early flowering in vitro, indicating that *AcFT*, at least when ectopically overexpressed, is able to promote flowering in kiwifruit. The unusual sequence not present in any other *FT* gene did not have a detrimental effect on the AcFT function. Rather, it enhanced its capacity to induce the flowering fate and terminate growth in tissue culture conditions, in agreement with the impaired growth and shoot tip abortion previously observed in transgenic *A. eriantha*. Regeneration resulting in in vitro floral initials and no viable plant development might have been the reason why only a small number of *A. eriantha* lines expressing low transgene levels have been recovered, and expression in kiwifruit flowers combined with in vitro flowering upon overexpression indicate that *AcFT* might also maintain the floral fate when expressed in flowers.

### 4.2 The *AcFT* promoter activity in Arabidopsis is restricted to the vascular tissue in a manner similar to the Arabidopsis *FT* promoter

The 3.5‐kb sequence upstream of the *AcFT* translation start site was sufficient to drive *GUS* expression in the vasculature of the distal half of cotyledons and the rosette leaves in Arabidopsis, before extending in the basipetal direction as the leaf ages. This is replicating the pattern of Arabidopsis *FT* promoter (Adrian et al., 2010; Takada & Goto, 2003) and is consistent with the basipetal pattern of dicot leaf development. The expression in Arabidopsis rosette leaves is comparable to expression profiles described in kiwifruit, where *AcFT* accumulated in basal leaves but was absent from terminal bud, distal leaves and root tissue (Varkonyi‐Gasic et al., 2013). On the other hand, differential expression recorded using *GUS* fused to a 3‐kb and 2.7‐kb sequence upstream of the *AcFT1* and *AcFT2* translation start sites, respectively, was consistent with *AcFT1* expression in kiwifruit terminal buds and *AcFT2* restriction to mature leaves (Voogd et al., 2017), suggesting that all promoter fragments in this study contained the regulatory elements required for specific expression patterns, although additional regulatory regions may exist outside these fragments.

Expression of *FT* in minor veins in the distal end of cotyledons and leaves was sufficient to promote flowering in Arabidopsis (Corbesier et al., 2007; Yoo, Hong, Jung, & Ahn, 2013). In minor veins, sucrose is loaded into the phloem, providing the osmotic potential difference required for bulk flow and long‐distance transport of phloem sap compounds (Sauer, 2007). The activity of *proAcFT* in minor veins during early development of Arabidopsis implies that the promoter might be regulated in similar manner in kiwifruit and that AcFT protein may be loaded into the vascular stream to perform a role outside its domain of expression. This is unlikely for the shoot tip‐associated *AcFT1* and less clear for *AcFT2*, which is expressed in kiwifruit mature leaves but potentially confined to major veins. Therefore, the role of *AcFT2* at native expression levels and domains needs to be studied further. Similarly, expression of *AcFT* in kiwifruit woody stem and dormant bud during winter chilling, which precedes spring bud break and flowering, cannot be addressed in Arabidopsis.

### 4.3 The *AcFT* promoter is active in Arabidopsis regardless of the photoperiod

Very little is known about the regulation of transcription of *FT* gene homologs in species other than Arabidopsis and rice. It has become obvious that while some commonalities exist, the regulatory mechanisms differ greatly between species (reviewed in Andres & Coupland, 2012). In spite of a remarkable similarity in expression domains detected with Arabidopsis *FT* and *AcFT* promoters in transgenic Arabidopsis, their activity in SD photoperiod was very different. In contrast to the Arabidopsis *FT* promoter with its day–length response (Adrian et al., 2010; Takada & Goto, 2003), *proAcFT* had similar activity under inductive and noninductive day–length conditions in Arabidopsis, providing the opportunity for functional studies in absence of endogenous FT.

The photoperiod‐dependent *FT* expression in Arabidopsis results from interaction with CO at the *cis*‐acting element in the *FT* proximal promoter (Tiwari et al., 2010) and elements in the distal promoter (Adrian et al., 2010). The CO binding site (Tiwari et al., 2010) has not been identified in *proAcFT* and the small increase in LUC activity detected in transactivation experiments could have resulted from the presence of multiple CCAAT box motifs, previously shown to bind the CO‐HAP3‐HAP5 complex to upregulate *FT* expression in Arabidopsis (Wenkel et al., 2006). In Arabidopsis, *FT* expression is repressed by MADS‐box proteins FLC and SVP as a result of binding to the CArG box motifs in the first intron and the promoter region (Lee et al., 2007; Li et al., 2008; Searle et al., 2006). Consistent with its small size (Varkonyi‐Gasic et al., 2013) and lack of CArG box motifs, the first intron did not affect expression of *AcFT*. Therefore, at this stage, it is unclear if *AcFT* interacts with kiwifruit SVP or FLC‐like MADS‐box proteins previously implicated in regulation of bud dormancy (Varkonyi‐Gasic et al., 2014; Wu et al., 2012, 2017).

### 4.4 AcFT is functional when expressed under its own promoter

Expression of *AcFT* cDNA under control of the 3.5‐kb *proAcFT* fragment was able to advance flowering in transgenic Arabidopsis Col‐0 in noninductive SD in a proportion of plants, suggesting that *AcFT* encoded an activator of flowering at native expression levels. The very early flowering confirmed that cotyledons were the main source of *AcFT* sufficient to induce bolting and flowering (Yoo et al., 2013). At later stages of development, *proAcFT* was insufficient to maintain flowering in noninductive conditions, leading to flower abortion and development of aerial rosettes with both Arabidopsis *FT* and *AcFT*, potentially reflecting the kiwifruit vegetative flushing that follows the first spring floral flush (Grant & Ryugo, 1982). Additionally, the fusion with GFP was capable of advancing flowering time, but the weak fluorescence prevented monitoring of the potential translocation of the AcFT:GFP fusion protein. Therefore, it cannot be distinguished if AcFT:GFP acts as a mobile signal, or affects other flower‐promoting mechanisms by acting locally.

In conclusion, *AcFT* promotes floral fate when overexpressed in kiwifruit, the combination of expression levels and protein activity of AcFT has the capacity to drive reproductive onset, while the conserved pattern in minor veins is consistent with vascular export and activity outside the domain of gene expression. Taken together, the data suggest that AcFT performs a role in orchestrating kiwifruit growth and flowering and is a candidate for kiwifruit florigen.

## CONFLICT OF INTEREST

The authors declare that the research was conducted in the absence of any commercial or financial relationships that could be construed as a potential conflict of interest.

## AUTHOR CONTRIBUTIONS

SM, JP, RH, and EV‐G conceived the research and designed the experiments. SM, TW, CV, LB, and RW performed the experiments. SM and EV‐G analyzed the data and wrote the manuscript. JP and AA revised the manuscript.
