# Supplementary material for: AcFT promotes kiwifruit in vitro flowering when overexpressed and Arabidopsis flowering when expressed in the vasculature under its own promoter
Source: Plant Direct. 2018 Jul 10;2(7):e00068. doi: 10.1002/pld3.68 (PMC6508797; doi:10.1002/pld3.68)
Supplement: Supplementary file 1 [file PLD3-2-e00068-s001.pdf]

Figure S1

(a)

```
 AcFT  ATGCCAAGACAGAGGGATCCTCTTGTTGTGGGGGGAGTAATAGGGGATGTTCTTGAACCCTTTGAAAGGTCTATAAATCTAAGGGTGACTTATAACAGCAGTAACAATAGTAATGGTAGGGAAATTACCAATGGGT
AcFT1  ATGCCTAGGGAGCGGGATCCTCTTGTAGTTGGGAGGGTGATAGGGGATGTTTTGGACCCCTTCACAAGGTCAATAGGGCTAAGGGTAACTTACAACA--A----------------TAAGGAAGTGAGCAATGGGT
AcFT2  ATGCCGAGGGATAGGGATCCTCTTGTAGTTGGGAGGGTGATAGGGGATGTTTTGGACCCCTTCACAAGGTCAATAGGACTAAGGGCAACTTACAACA--A----------------TAAGGAAGTGAACAATGGGT

 AcFT  GTGAGCTTAGGCCTTCACAAGTTGTCAACCAACCGAGGGTTGATATTGGTGGCGATGATCTTAGGACCTTTTACACTCTTGTGATGGTGGACCCTGATGCTCCCAGCCCAAGTGACCCAAGCCTTAGGGAATACTT
AcFT1  GTGAGCTCAAGCCTTCCCAAGTTGTCAACCAACCTAGAGTTGATGTTGGAGGTGATGACCTTAGGACGTTCTATACTCTTGTTATGGTGGACCCTGATGCTCCCAGTCCAAGTAATCCAAACCTAAGGGAGTACTT
AcFT2  GTGAGTTCAAGCCTTCCCAAGTTGTCAACCAACCTAGGGTTGATATTGGAGGTGATGACCTAAGGACCTTCTATACCCTTGTTATGGTGGACCCTGATGCTCCCAGTCCAAGTGATCCAAACCTGAGGGAGTACTT

 AcFT  GCATTGGTTGGTGACAGATATTCCAGCAACTACAGGAGCAAGCTTTGGACAAGAGGTGGTGTATTATGAAAGCCCAAGGCCATCCGTAGGAATCCATCGCTTTGTTCTGGTGCTATTTCGACAGTTGGGAAGACAG
AcFT1  GCATTGGTTGGTGACGGATATCCCAGCAACCACAGGAGTAAGTTTTGGACAGGAGGTGGTTTGTTACGAGAGCCCTCGACCATCAATGGGTTTTCATCGCTTTGTGTTCGTGTTGTTTCGACAACTGGGCCGCCAG
AcFT2  GCATTGGTTGGTGACTGATATTCCAGCAACCACAGGAGTAAGCTTTGGACAAGAGGTGGTTTGTTACGAGAACCCTCAACCATTCATGGGGATTCATCGCTTTGTGTTCGTGTTGTTCCGGCAATTGGGTCGCCAG

 AcFT  ACAGTTTATGCACCAGGTTGGCGCCAGAATTTCAACACTAGGGACTTTGCTGAGCTTTACAATCTTGGTGTCCCTGTTGCTGCTATATATTTCAACTGCCAGAGGGAGACTGGGTCTGGAGGGAGAAGACGATGA
AcFT1  ACTGTTTATGCTCCAAGGTGGCGCCAGAATTTTAACACCAGGGATTTTGCCGAGCTTTATAATCTTGGCTCTCCAGTTGCTGCCGTCTTCTTTAACGGCCAGAGGGAGAGCGGCTCTGGTGGGCGAAGACGCTGA
AcFT2  ACAGTGTATCCTCCAGGGTGGCGCCAGAATTTTAACACTAGGTACTTTGCCGAGCTTTACAATCTTGGCTCTCCAGTTGCTGCCGTCTACTTTAACTGCCAGAGAGAGAGTGGTTCTGGAGGGCGAAGATGCTGA
```

(b)

```
           Actinidia_chinensis_FT_JX417423 ---MPRQRDPLVVGGVIGDVLEPFERSINLRVTYNSSNNSNGREITNGCELRPSQVVNQPRVDIGGDDLRTFYTLVMVDPDAPSPSDPSLREYLHWLVTD
          Actinidia_chinensis_FT1_KX611594 ---MPRERDPLVVGRVIGDVLDPFTRSIGLRVTYNN------KEVSNGCELKPSQVVNQPRVDVGGDDLRTFYTLVMVDPDAPSPSNPNLREYLHWLVTD
          Actinidia_chinensis_FT2_KX611595 ---MPRDRDPLVVGRVIGDVLDPFTRSIGLRATYNN------KEVNNGCEFKPSQVVNQPRVDIGGDDLRTFYTLVMVDPDAPSPSDPNLREYLHWLVTD
         Arabidopsis_thaliana_FT_At1g65480 --MSINIRDPLIVSRVVGDVLDPFNRSITLKVTYGQ------REVTNGLDLRPSQVQNKPRVEIGGEDLRNFYTLVMVDPDVPSPSNPHLREYLHWLVTD
       Arabidopsis_thaliana_TSF_At4g20370 --MSLSRRDPLVVGSVVGDVLDPFTRLVSLKVTYGH------REVTNGLDLRPSQVLNKPIVEIGGDDFRNFYTLVMVDPDVPSPSNPHQREYLHWLVTD
Solanum_lycopersicum_SP3D/SFT_AY186735 ---MPRERDPLVVGRVVGDVLDPFTRTIGLRVIYRD------REVNNGCELRPSQVINQPRVEVGGDDLRTFFTLVMVDPDAPSPSDPNLREYLHWLVTD
           Solanum_tuberosum_FT_GU223211 ----MPRVDPLIVGRVIGDVLDPFTRSVDLRVVYNN------KDVNNACVLKPSQVVMQPRVHIGGDDLRNFYTLIMVDPDAPSPSNPDLREYLHWLVTD
             Vitis_vinifera_FT_DQ871590 ---MPRERDPLVVGRVVGDVLDPFLRSITLRVTYNN------REVANGCEFRPSQLVSQPRVDIGGDDLRTFYTLVMVDPDAPSPSNPNLREYLHWLVTD
        Cucurbita_moschata_FTL1_ABR20498 ---MPRDRDPLVVGRVIGDVIDSFSRSISIRVAYNS------RKVKNGCELKPCQVINKPRVEIGGTDLRTFFTLVMVDPDAPSPSDPNLREYLHWLVTD
        Cucurbita_moschata_FTL2_ABR20499 ---MPRDRDPLVIGRVIGDVIDSFTRSISIRATYNN------REISNGCELKPSQVVNQPRVEIGGTDLRTFFTLVMVDPDAPSPSDPNLREYLHWLVTD
          Populus_deltoides_FT1_AAS00056 ---MPRDREPLSVGRVIGDVLDPFTRSISLRVNYNS------REVNNGCELKPSHVVNQPRVDIGGEDLRTFYTLVMVDPDAPSPSNPNLREYLHWLVTD
          Populus_deltoides_FT2_AY515152 ---MPRDREPLSVGRVIGDVLDPFTRSISLRVNYNS------REVNNGCELKPSHVVNQPRVDIGGEDLRTFYTLVMVDPDAPSPSNPNLREYLHWLVTD
      Medicago_truncatula_FTa1_HQ721813 --MAGSSRNPLAVGRVIGDVIDSFENSIPLRVTYGN------RDVNNGCELKPSQIGNQPRVSVGGNDLRNLYTLVMVDPDSPSPSNPTFKEYLHWLVTD
           Pisum_sativum_FTa1_HQ538822 --MAGSSRNPLAVGRVIGDVIDPFENSVPLRVTYGS------RDVNNGCELKPSHVGNQPRVNVGGNDLRNIYTLVLVDPDSPSPSNPTFREYLHWLVTD
            Citrus_unshiu_FT_AB027456 --MSSRERDPLIVGRVVGDVLDNFTRTIPMRITYSN------KDVNNGRELKPSEVLNQPRAEIGGDDLRTFYTLVMVDPDAPSPSDPSLREYLHWLVTD
          Malus_domestica_FT1_AB161112 ---MPRDRDPLVVGRVVGDVLDPFTRSVSLRVTYGT------KEVNNGCELKPSEVVQQPRADIGGDDLRTFYTLVMVDPDAPSPSDPNLKEYLHWLVTD
          Malus_domestica_FT2_AB458504 ---MPRDRDPLVVGRVVGDVLDPFTRSVSLRVTYGN------KEVNNGCELKPSQVVHQPRVDTGGDDLRTFYTLVMVDPDAPSPSDPNLKEYLHWLVTD
                          OsHd3a_Oryza MAGSGRDRDPLVVGRVVGDVLDAFVRSTNLKVTYGS------KTVSNGCELKPSMVTHQPRVEVGGNDMRTFYTLVMVDPDAPSPSDPNLREYLHWLVTD

           Actinidia_chinensis_FT_JX417423 IPATTGASFGQEVVYYESPRPSVGIHRFVLVLFRQLGRQTVYAPGWRQNFNTRDFAELYNLGVPVAAIYFNCQRETGSGGRRR------
          Actinidia_chinensis_FT1_KX611594 IPATTGVSFGQEVVCYESPRPSMGFHRFVFVLFRQLGRQTVYAPRWRQNFNTRDFAELYNLGSPVAAVFFNGQRESGSGGRRR------
          Actinidia_chinensis_FT2_KX611595 IPATTGVSFGQEVVCYENPQPFMGIHRFVFVLFRQLGRQTVYPPGWRQNFNTRYFAELYNLGSPVAAVYFNCQRESGSGGRRC------
         Arabidopsis_thaliana_FT_At1g65480 IPATTGTTFGNEIVCYENPSPTAGIHRVVFILFRQLGRQTVYAPGWRQNFNTREFAEIYNLGLPVAAVFYNCQRESGCGGRRL------
       Arabidopsis_thaliana_TSF_At4g20370 IPATTGNAFGNEVVCYESPRPPSGIHRIVLVLFRQLGRQTVYAPGWRQQFNTREFAEIYNLGLPVAASYFNCQRENGCGGRRT------
Solanum_lycopersicum_SP3D/SFT_AY186735 IPATTGSSFGQEIVSYESPRPSMGIHRFVFVLFRQLGRQTVYAPGWRQNFNTRDFAELYNLGLPVAAVYFNCQRESGSGGRRRSAD---
           Solanum_tuberosum_FT_GU223211 IPATTNTSFGNEVVCYENPTPTMGIHRYVLVLFRQLRRETVYAPGWRQNFNTRDFAELYNLGLPVAAVYFNCHRESGTGGRRR
             Vitis_vinifera_FT_DQ871590 IPATTGANFGQEIVCYESPRPTAGIHRFVFVLFRQLGRQTVYAPGWRQNFNTRDFAELYNLGLPVAAVYFNCQREGGSGGRRS------
        Cucurbita_moschata_FTL1_ABR20498 IPATTEATFGQEIVCYENPRPTAGIHRFVLVLFRQLGRQTVYAPGWRQNFNTRHFAELYNLGSPVAAVYFNCQRENGSGGRRRAGDECS
        Cucurbita_moschata_FTL2_ABR20499 IPATTGATFGQEIVCYESPRPTVGIHRLVLVLFRQLGRQTVYAPGWRQNFNTRDFAELYNLGLPVAAVYFNCQRESGSGGRRRSQDDF-
          Populus_deltoides_FT1_AAS00056 IPATTGANFGQEVVCYESPRPTAGIHRFVFVLFRQLGRQTVYPPGWRQNFNTRDFAELYNLGSPVAAVYFNCQRESGSGGRRP------
          Populus_deltoides_FT2_AY515152 IPATTGANFGQEVVCYESPRPTAGIHRFVFVLFRQLGRQTVYPPGWRQNFNTRDFAELYNLGSPVAAVYFNCQRESGSGGRRP------
      Medicago_truncatula_FTa1_HQ721813 IPGTTEVTFGNEVVNYERPRPTSGIHRFVFVLFRQQCRQRVYAPGWRQNFNTREFAELYNLGSPVAAVFFNCQRESGSGGRTFR-----
           Pisum_sativum_FTa1_HQ538822 IPATTEVSFGNEIVSYERPRPTSGIHRFVFILFRQQCRQRVYAPGWRQNFNTREFAELYNLGSPVAAVFFNCQRESGSGGRTFR-----
            Citrus_unshiu_FT_AB027456 IPATTGASFGQEIVNYESPRPTMGIHRFVFVLFRQLGRQTVYAPGWRQNFSTRDFAELYNLGPPVAAVYFNCQRESGSGGRPVRR----
          Malus_domestica_FT1_AB161112 IPATTAASFGQEIVCYESPRPTVGIHRFVLVVFRQLGRQTVYAPGWRQNFNTRDFAELYNLGLPVSVVYFNCQREGGSGGRRR------
          Malus_domestica_FT2_AB458504 IPATTAASFGQEIVCYESPRPTVGIHRFVFVLFRQLGRQTVYAPGWRQNFNTRDFAELYNLGLPVAAVYFNCQRESGSGGRRR------
                          OsHd3a_Oryza IPGTTAASFGQEVMCYESPRPTMGIHRLVFVLFQQLGRQTVYAPGWRQNFNTKDFAELYNLGSPVAAVYFNCQREAGSGGRRVYP----
```

Supplementary Figure S1. AcFT harbours an unusual short fragment. (a) Alignment of kiwifruit *FT*-like coding sequences. (b) Alignment of FT-like protein sequences from diverse plants.
