# Supplementary material for: AcFT promotes kiwifruit in vitro flowering when overexpressed and Arabidopsis flowering when expressed in the vasculature under its own promoter
Source: Plant Direct. 2018 Jul 10;2(7):e00068. doi: 10.1002/pld3.68 (PMC6508797; doi:10.1002/pld3.68)
Supplement: Supplementary file 2 [file PLD3-2-e00068-s002.pdf]

Figure S2

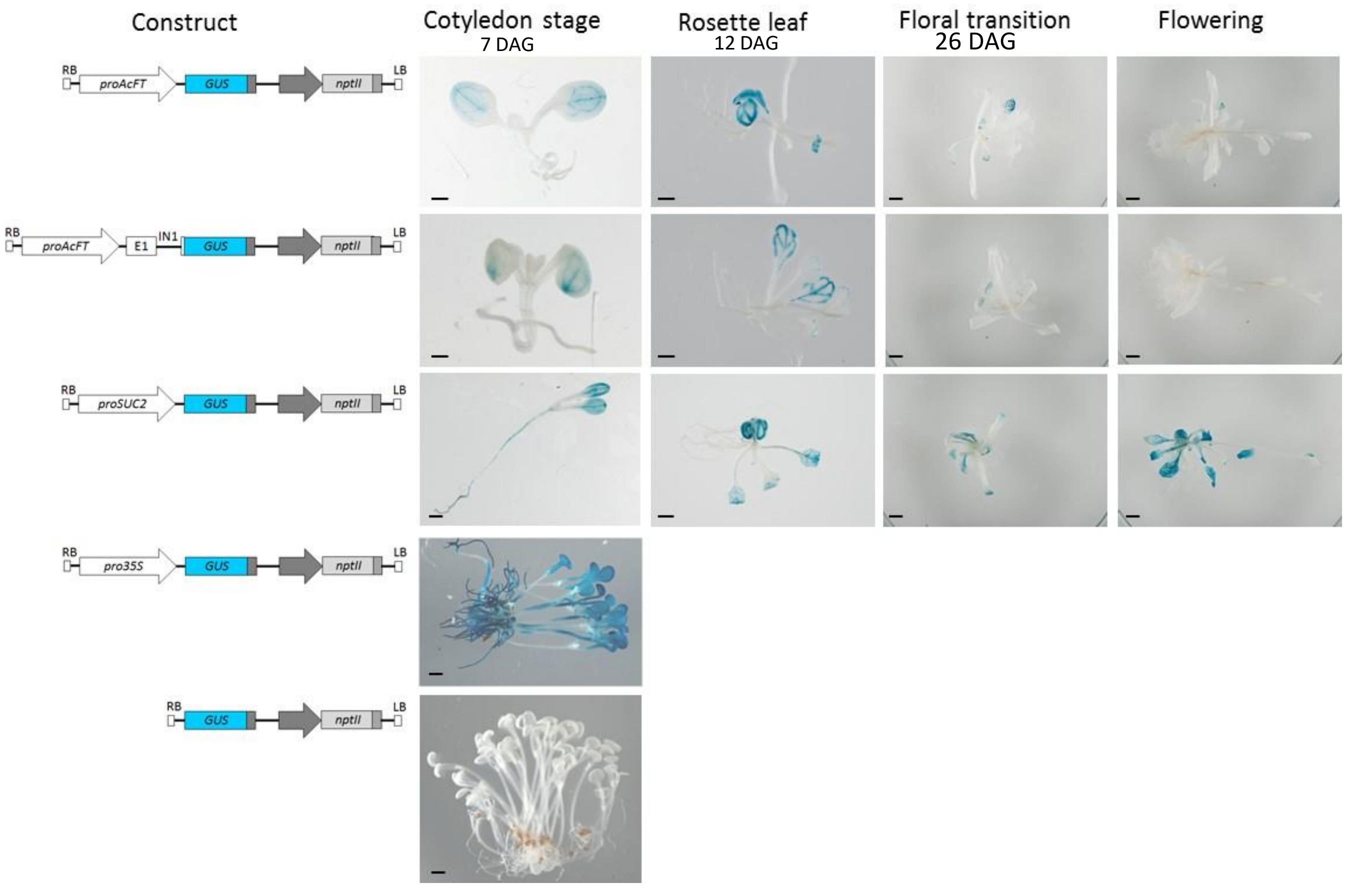

Supplementary Figure S2. Histochemical localization of GUS activity during development of transgenic Arabidopsis plants grown in long day conditions. Promoter fusions with *uidA* (*GUS*) reporter gene are presented as schematics: 3.5 kb sequence upstream of the *AcFT* translation start sites (*proAcFT*), a fragment containing *proAcFT*, *AcFT* first exon (E1), first intron (IN1) and the first eight codons of the second exon, the *SUCROSE TRANSPORTER 2* (*SUC2*) promoter (*proSUC2*), CaMV *35S* promoter (*pro35S*), and a promoterless control. DAG, days after germination.
