# Supplementary material for: AcFT promotes kiwifruit in vitro flowering when overexpressed and Arabidopsis flowering when expressed in the vasculature under its own promoter
Source: Plant Direct. 2018 Jul 10;2(7):e00068. doi: 10.1002/pld3.68 (PMC6508797; doi:10.1002/pld3.68)
Supplement: Supplementary file 3 [file PLD3-2-e00068-s003.pdf]

Figure S3

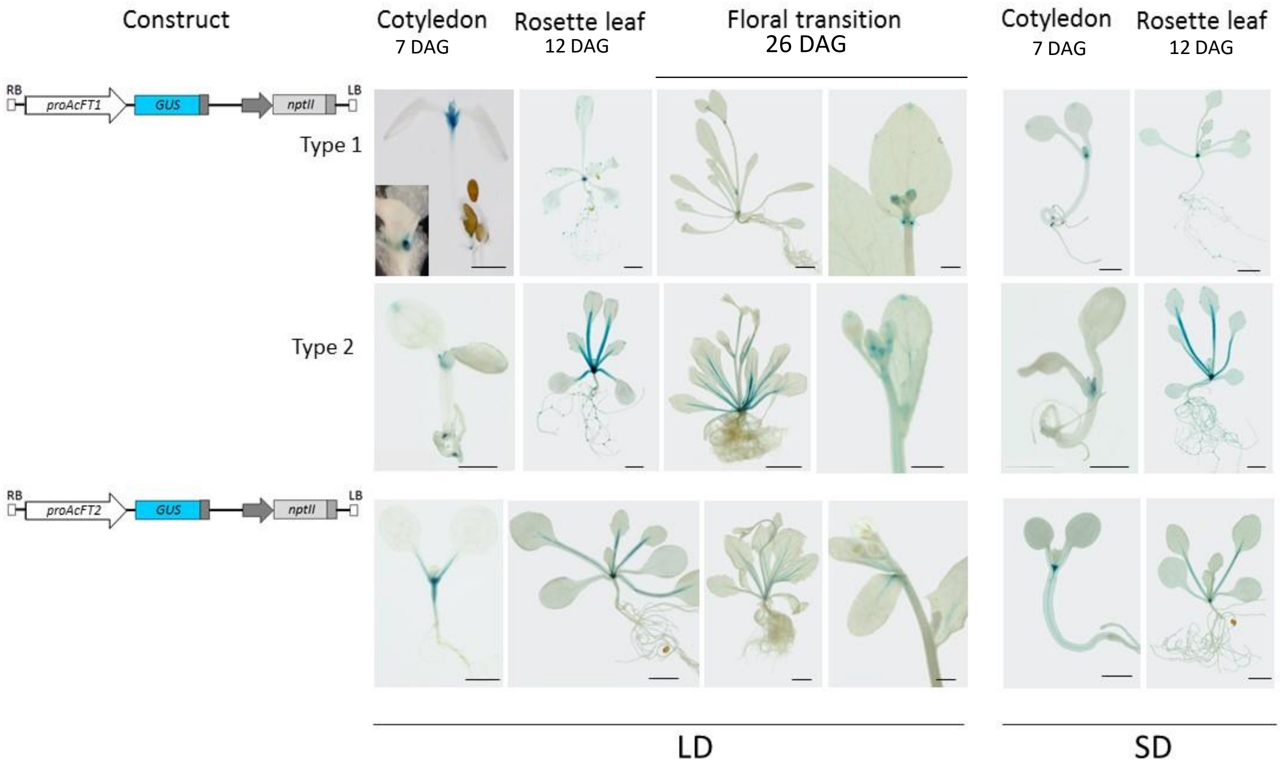

Supplementary Figure S3. Histochemical localization of GUS activity during development of transgenic Arabidopsis plants grown in long day (LD) and short day (SD) conditions. *AcFT1* and *AcFT2* promoter fusions with *uidA* (*GUS*) reporter gene are presented as schematics. DAG, days after germination.
