# Supplementary figures and images for: AcFT promotes kiwifruit in vitro flowering when overexpressed and Arabidopsis flowering when expressed in the vasculature under its own promoter
Source: Plant Direct. 2018 Jul 10;2(7):e00068. doi: 10.1002/pld3.68 (PMC6508797; doi:10.1002/pld3.68)

Figure S4

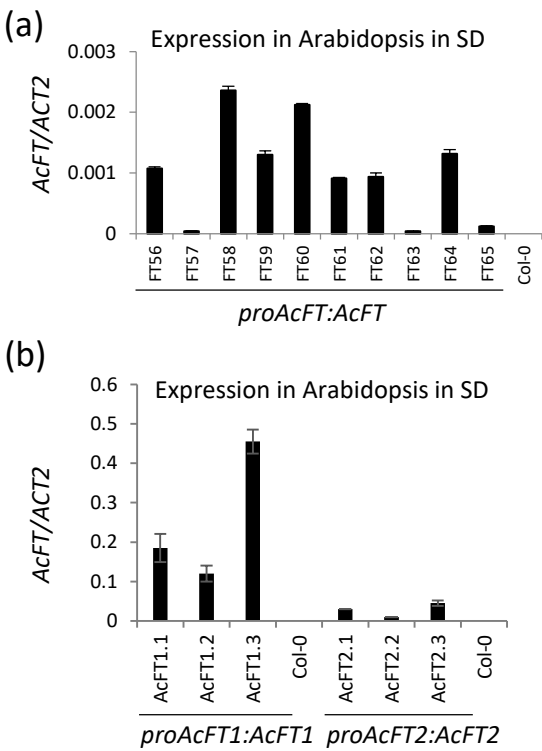

Supplementary Figure S4. Relative expression  $\pm$  SE in transgenic Arabidopsis.

Supplement: Supplementary file 4 [file PLD3-2-e00068-s004.pdf]
