# Supplementary material for: AcFT promotes kiwifruit in vitro flowering when overexpressed and Arabidopsis flowering when expressed in the vasculature under its own promoter
Source: Plant Direct. 2018 Jul 10;2(7):e00068. doi: 10.1002/pld3.68 (PMC6508797; doi:10.1002/pld3.68)
Supplement: Supplementary file 5 [file PLD3-2-e00068-s005.pdf]

Table S1. Oligonucleotide primers used in this study.

#	Primer name	Primer sequence (5' to 3')	Purpose
1	AcFT-IPCR-F	ATGCCAAGACAGAGGGATCCTC	inverse PCR
2	AcFT-IPCR-R	CAATATCAACCCTCGGTTGGT	inverse PCR
3	proAcFT-F	AAGCTTACTTTTTTCATTTTATAGTCT	proAcFT amplification
4	proAcFT-R	CACTCCCTCTCTCCTCAAAACTA	proAcFT amplification
5	FTIN1-R	GGGAGCATCAGGGTCCACCAT	proAcFT::IN1
6	EcoRI-SpeI-AcFT-F	CCCCCCGAATTCACTAGTAACAATGCCAAGACAGAGGGAT	AcFT:GFP fusion
7	AcFT-GS-R	GCTTGAGCCAGAGCCAGAGCCACCTCGTCTTCTCCCTCCAGA	AcFT:GFP fusion
8	GS-GFP-F	GGTGGCTCTGGCTCTGGCTCAAGCGTGAGCAAGGGCGAGGAG	AcFT:GFP fusion
9	GFP-XhoI-XbaI-R	CCCCCCTCTAGACTCGAGTTACTTGTACAGCTCGTCC	AcFT:GFP fusion
10	proAcFT-F-SacI	GGGAGCTCAAGCTTACTTTTTTCATTTTATA	proAcFT:GUS, proAcFT::IN1:GUS
11	proAcFT-R-EcoRI	TGAATTCCCCATGGCTCCCTCTCTCCTCA	proAcFT:GUS
12	FTIN1-R-EcoRI	ATGAATTCGGGAGCATCAGGGTCCACCAT	proAcFT::IN1:GUS
13	proSUC2-F-SacI	CCGAGCTCCAAAATAGCACACCA	proSUC2:GUS
14	proSUC2-R-SpeI	CCACTAGTGTTACAAACCAAGA	proSUC2:GUS
15	AcFT-F-EcoRI	CGGAATTCACTAGTATGCCAAGAC	proAcFT:AcFT
16	AcFT-R-ClaI	CCCATCCGATCATCGTCTTCTC	proAcFT:AcFT
17	AcFT-F-NcoI-EcoRI-	CCCCCATGGGAATTCACTAGTATGTCTATAAATATAAGAGA	proAcFT:FT
18	AcFT-R_XbaI	CCCCTCTAGACTAAAGTCTTCTTCCTCCGCAG	proAcFT:FT
19	proAcFT-F-HindIII	GGAAGCTTACTTTTTTCATTTTATAGTCT	proAcFT:LUC
20	proAcFT-R-NcoI	CACCATGGCTCCCTCTCTCCTCAAAACTA	proAcFT:LUC
21	proFT-F-NotI	ATCTGCGGCCGCACTAACATGATTG	proFT:LUC
22	proFT-R-NcoI	GTCTTCCATGGATCTTGAACAAACA	proFT:LUC
23	proAcFT1-F	CTCACTGACACAGCAATCAGT	proAcFT1 amplification
24	proAcFT1-R	AATACTACTCTATATTATA	proAcFT1 amplification
25	proAcFT2-F	CAAATCATTCTAATCTTGA	proAcFT2 amplification
26	proAcFT2-R	AACACGACTCTATATTATA	proAcFT2 amplification
27	pAcFT1-F-AscI	ATGGCGCGCCCTCACTGACACAGCAATCAGT	proAcFT1:GUS
28	pAcFT1-R-KpnI	ACGGTACCAATACTACTCTATATTATA	proAcFT1:GUS
29	pAcFT2-F-AscI	ATGGCGCGCCCAAATCATTCTAATCTTGA	proAcFT2:GUS
30	pAcFT2-R-KpnI	ACGGTACCAACACGACTCTATATTATA	proAcFT2:GUS
31	AcFT-F	CCAACCGAGGGTTGATATTG	RT-qPCR
32	AcFT-R	GCTTGCTCCTGTAGTTGCTGGA	RT-qPCR
33	AcFT1-F	CTCTCCAGTTGCTGCCGTCTT	RT-qPCR
34	AcFT1-R	TCCCAACATAATACGGTCGCACT	RT-qPCR
35	AcFT2-F	CTCTCCAGTTGCTGCCGTCTA	RT-qPCR
36	AcFT2-R	TGTGCCTTACTAGTCCAAACATCG	RT-qPCR
37	AtACT2-F	CTCTCCCGCTATGTATGTCGCCA	RT-qPCR
38	AtACT2-R	GTGAGACACACCATCACCAG	RT-qPCR
39	GUS-F	GCGATTTGGAAACGGCAGAG	RT-qPCR
40	GUS-R	ATTGAGTGCAGCCCGGCTAA	RT-qPCR
41	AdACTIN-F	CCAAGGCCAACAGAGAGAAG	RT-qPCR
42	AdACTIN-R	GACGGAGGATAGCATGAGGA	RT-qPCR
