# Supplementary material for: AcFT promotes kiwifruit in vitro flowering when overexpressed and Arabidopsis flowering when expressed in the vasculature under its own promoter
Source: Plant Direct. 2018 Jul 10;2(7):e00068. doi: 10.1002/pld3.68 (PMC6508797; doi:10.1002/pld3.68)
Supplement: Supplementary file 6 [file PLD3-2-e00068-s006.pdf]

Table S2. Constructs used in this study.

Purpose	Constructs	Method	Primer #
Cloning and sequencing	pGEM iPCR *gAcFT* fragment	Inverse PCR fragement cloned into pGEM-T Easy (Promega)	1, 2
	pGEM *proAcFT*	3.5 kb *proAcFT* (including 5' UTR) amplified and cloned into pGEM-T Easy	3, 4
	pGEM *proAcFT::IN1*	Genomic fragment containing 3.5 kb *proAcFT* , first exon, first intron and the first eight codons of the second exon	3, 5
	pUC19 *AcFT:GFP*	*AcFT* and *GFP* amplified separately to include the glycine-serine linker and subjected to overlap amplification*	6, 7, 8, 9
GUS fusions	pHEX14 *proAcFT* :*GUS*	*proAcFT* amplified and cloned as SacI-EcoRI fragment into the pHEX14 binary vector	10, 11
	pHEX14 *proAcFT* ::*IN1* :*GUS*	*proAcFT::IN1* amplified and cloned cloned as SacI-EcoRI fragment into the pHEX14 binary vector	10, 12
	pHEX14 *proSUC2:GUS*	*proSUC2* (Stadler et al., 2005) amplified and cloned as SacI-SpeI fragment into the pHEX14 binary vector	13, 14
	pHEX14 (promoterless GUS)	Hellens et al., 2005	
	pHEX4 *pro35S:GUS*	Hellens et al., 2005	
AcFT vectors	pHEX14 *proAcFT:AcFT*	*AcFT* amplified and cloned as EcoRI-ClaI fragment to replace *GUS* in pHEX14 *proAcFT:GUS*	15, 16
	pHEX14 *proAcFT:FT*	*FT* amplified and cloned to replace *AcFT* in pHEX14 *proAcFT:AcFT*	17, 18
	pSAK778 *proSUC2:AcFT*	Varkonyi-Gasic et al., 2013	
	pSAK778 *pro35S:AcFT*	Varkonyi-Gasic et al., 2013	
GFP fusions*	pHEX14 *proAcFT* :*AcFT:GFP*	*AcFT:GFP* subcloned as EcoRI/XbaI fragment into pHEX14 *proAcFT:AcFT* (to replace *AcFT*)	
	pSAK778 *proSUC2:AcFT:GFP*	*AcFT:GFP* subcloned as SpeI/XhoI fragment into pSAK778 *proSUC2:AcFT* (to replace *AcFT*)	
LUC fusions	pGreen *proAcFT:LUC*	*proAcFT* amplified and cloned as a HindIII-NcoI fragment into pGreenII 0800-LUC binary vector	19, 20
	pGreen *proFT:LUC*	1.8 kb fragment of Arabidopsis *FT* promoter amplified and cloned as a NotI-NcoI fragment into pGreenII 0800-LUC	21, 22
AcFT1 and *AcFT2* constructs	pGEM *proAcFT1*	3 kb *proAcFT1* (including 5' UTR) amplified and cloned into pGEM-T Easy	23, 24
	pGEM *proAcFT2*	2.7 kb *proAcFT2* amplified and cloned into pGEM-T Easy	25, 26
	pHEX14 *proAcFT1* :*GUS*	*proAcFT1* amplified and cloned as AscI-KpnI fragment into the pHEX14 binary vector	27, 28
	pHEX14 *proAcFT2* :*GUS*	*proAcFT2* amplified and cloned as AscI-KpnI fragment into the pHEX14 binary vector	29, 30
	pUC19 *AcFT1*	Voogd et al, 2017	
	pUC19 *AcFT2*	Voogd et al, 2017	
	pHEX14 *proAcFT1:AcFT1*	*AcFT1* cloned as KpnI-HindIII fragment to replace *GUS* in pHEX14 *proAcFT1:GUS*	
	pHEX14 *proAcFT2:AcFT2*	*AcFT2* cloned as KpnI-XbaI fragment to replace *GUS* in pHEX14 *proAcFT2:GUS*	
	pHEX14 *proAcFT1:AcFT2*	*AcFT2* cloned as KpnI-XbaI fragment to replace *GUS* in pHEX14 *proAcFT1:GUS*	

*To generate fusion constructs with green fluorescent protein gene (*GFP*), AcFT coding sequence (stop codon excluded) was amplified with specific oligonucleotide primers to include EcoRI-SpeI restriction sites at the 5’ terminus and the glycine-serine linker sequence at the 3’ terminus. The enhanced GFP sequence was amplified from pHEX2S:GFP to include the 5’ terminal glycine-serine linker and XhoI-XbaI at the 3’ terminus. pHEX2S:GFP is a derivative of pHEX2 (Hellens et al., 2005) designed previously to include the *35S* promoter driven GFP(S65T) (Heim et al., 1995). Both amplification products were combined after purification and subjected to overlap amplification using the EcoRI-SpeI-AcFT and GFP-XhoI-XbaI oligonucleotide primers to generate the *AcFT* :*GFP* fusion, which was cloned as EcoRI/XbaI into pUC19.
