# Supplementary material for: AcFT promotes kiwifruit in vitro flowering when overexpressed and Arabidopsis flowering when expressed in the vasculature under its own promoter
Source: Plant Direct. 2018 Jul 10;2(7):e00068. doi: 10.1002/pld3.68 (PMC6508797; doi:10.1002/pld3.68)
Supplement: Supplementary file 7 [file PLD3-2-e00068-s007.pdf]

Dear Editors,

Thank you for considering our manuscript for publication. We would like to thank both reviewers for suggestions and constructive criticism.

We carefully revised our manuscript according to the reviewers' comments and suggestions. In particular, we have: included a description of *Actinidia* phenology in Introduction, explained Figures 2 and S2, clarified the graph and removed non-essential data (previously not shown but mentioned in text) in Figure 4b, as requested by Reviewer 1. We uploaded Figure 3 again, hoping to solve the possible pdf conversion problem identified by Reviewer 2.

Please find our response to reviewers' comments below.

A manuscript with tracked changes is uploaded as a supplementary file.

I hope the revised manuscript meets with your approval.

Kind regards,

Erika Varkonyi-Gasic

Reviewer #1:

This manuscript showed FT-mediated regulation of growth and flowering in kiwifruit using the own and/or Arabidopsis promoters combined with several FT genes of kiwifruit/Arabidopsis under flowering inductive and non-inductive conditions. The subject falls within the general scope of the journal, and the article is a new and original contribution. The interpretations and conclusions are sound enough and are justified by the data, and are consistent with the objectives. The title clearly reflects the contents of the paper and the abstract is sufficiently informative and can be read in isolation. Therefore, the manuscript contains new interesting insights to warrant publication. Before acceptance, try to address the below minor questions/suggestions.

1) In natural field condition, when does floral induction (or visible floral initiation) take place in kiwifruit? The authors focused on day length (SD/LD) in this study; how about the involvement of temperature in flower induction? Please briefly describes the basic phenology in kiwifruit flowering.

- We have included a paragraph that briefly describes kiwifruit phenology. Temperature (cold, i.e. winter chilling) is indeed essential for kiwifruit flowering, whilst photoperiod (the shortening of the day-length) drives growth cessation and budset (reference describing this included). The reason for focusing on photoperiod was the work we performed in Arabidopsis, where we wanted to be able to maintain the 'non-flowering' state of our model. The activity of AcFT promoter in conditions when endogenous FT is not expressed in Arabidopsis provided the opportunity to test if "native" expression levels and domains have the ability to drive flowering. We could not perform the chilling work in Arabidopsis; as a herbaceous plant, it would not survive the conditions that drive budbreak and flowering in kiwifruit.

2) Provide information for "E1" and "IN1" in Figs, 2 and S2. What did they mean?

- The first exon (exon 1) and the first intron (intron 1), now described in figure legend.

3) The authors stated in lines 221-223, "A translational fusion including the AcFT first exon, first intron and the first eight codons of the second exon was also evaluated to examine whether regulatory regions exist in the first intron". Which data (Fig. S2?) corresponds to this experiment? If Fig. S2, please clearly indicate the experimental details in figure legend of Figure S2.

- It is now described

4) The authors stated in lines 283-285, "Four out of seven lines flowered early in SD conditions, after the plants produced between 10 and 16 leaves, in contrast to >30 in control lines (Figure 4b)". It was hard to count the line numbers in the present figure; for me, it looks "three out of six lines".

- The figure presents the range, if more than one plant flowered at the same time, they are presented as a single dot (this is now described in the legend). We wanted to show that our promoter was capable to deliver sufficient Arabidopsis FT to drive flowering in Arabidopsis (in conditions when the Arabidopsis FT promoter is inactive and all control plants flower late). But an unintentional mistake has been made, it is three rather than four out of seven lines (now corrected). We have increased the size of the graph in Figure 4b for easier interpretation.

5) The authors stated in lines 290-292, "All Col-0 proAcFT:AcFT plants flowered in LD after the plants produced between 7 and 10 leaves, whilst ten Col-0 controls flowered after producing 8-10 leaves". Figure showed the results in SD condition; which data are comparable to this experiment in LD? Data not shown?

- Data not shown. As non-essential, now removed from the text.

Reviewer #2:

This is an interesting and well-done piece of work and manuscript. I have no substantial suggestions. In my copy of the manuscript, Figure 3 is not in English. I have no idea what language it is in.

- The pdf file on my screen seems normal and the legend is English, but copy-paste in Word converts it in random symbols: ŝŐƵƌĞ ϯ dŚĞ Ð&dÐƌŽŵŽƚĞƌ ŝƐ ŶŽƚ ƐƚƌŽŶŐůLJ ƌĞŐƵůĂƚĞĚ ďLJ ÐŚŽƚŽÐĞƌŝŽĚ ŝŶ ƚƌĂŶƐŐĞŶŝÐ ...
- Copy-paste of other figure legends is fine. I do not understand why, but to solve this problem, we resubmitted the Figure 3 pdf.
